# Adrenal-permissive *HSD3B1* genetic inheritance and risk of estrogen-driven postmenopausal breast cancer

**DOI:** 10.1172/jci.insight.150403

**Published:** 2021-10-22

**Authors:** Megan L. Kruse, Mona Patel, Jeffrey McManus, Yoon-Mi Chung, Xiuxiu Li, Wei Wei, Peter S. Bazeley, Fumihiko Nakamura, Aimalie Hardaway, Erinn Downs, Sarat Chandarlapaty, Mathew Thomas, Halle C.F. Moore, George T. Budd, W.H. Wilson Tang, Stanley L. Hazen, Aaron Bernstein, Serena Nik-Zainal, Jame Abraham, Nima Sharifi

**Affiliations:** 1 Department of Hematology and Oncology, Taussig Cancer Institute;; 2 GU Malignancies Research Center, Department of Cancer Biology, Lerner Research Institute;; 3 Cancer Biostatistics Section, Taussig Cancer Institute,; 4 Department of Quantitative Health Sciences, Lerner Research Institute; and; 5 Pathology and Laboratory Medicine Institute, Cleveland Clinic, Cleveland, Ohio, USA.; 6 Human Oncology and Pathogenesis Program, Memorial Sloan Kettering Cancer Center, New York, New York, USA.; 7 Department of Cardiovascular and Metabolic Sciences, Lerner Research Institute, and Heart, Vascular and Thoracic Institute, Cleveland Clinic, Cleveland, Ohio, USA.; 8 Academic Department of Medical Genetics, School of Clinical Medicine, University of Cambridge, Cambridge, United Kingdom.

**Keywords:** Endocrinology, Oncology, Breast cancer, Prostate cancer, Sex hormones

## Abstract

**BACKGROUND:**

Genetics of estrogen synthesis and breast cancer risk has been elusive. The 1245A→C missense-encoding polymorphism in *HSD3B1*, which is common in White populations, is functionally adrenal permissive and increases synthesis of the aromatase substrate androstenedione. We hypothesized that homozygous inheritance of the adrenal-permissive *HSD3B1*(1245C) is associated with postmenopausal estrogen receptor–positive (ER-positive) breast cancer.

**METHODS:**

A prospective study of postmenopausal ER-driven breast cancer was done for determination of *HSD3B1* and circulating steroids. Validation was performed in 2 other cohorts. Adrenal-permissive genotype frequency was compared between postmenopausal ER-positive breast cancer, the general population, and postmenopausal ER-negative breast cancer.

**RESULTS:**

Prospective and validation studies had 157 and 538 patients, respectively, for the primary analysis of genotype frequency by ER status in White female breast cancer patients who were postmenopausal at diagnosis. The adrenal-permissive genotype frequency in postmenopausal White women with estrogen-driven breast cancer in the prospective cohort was 17.5% (21/120) compared with 5.4% (2/37) for ER-negative breast cancer (*P* = 0.108) and 9.6% (429/4451) in the general population (*P* = 0.0077). Adrenal-permissive genotype frequency for estrogen-driven postmenopausal breast cancer was validated using Cambridge and The Cancer Genome Atlas data sets: 14.4% (56/389) compared with 6.0% (9/149) for ER-negative breast cancer (*P* = 0.007) and the general population (*P* = 0.005). Circulating androstenedione concentration was higher with the adrenal-permissive genotype (*P* = 0.03).

**CONCLUSION:**

Adrenal-permissive genotype is associated with estrogen-driven postmenopausal breast cancer. These findings link genetic inheritance of endogenous estrogen exposure to estrogen-driven breast cancer.

**FUNDING:**

National Cancer Institute, NIH (R01CA236780, R01CA172382, and P30-CA008748); and Prostate Cancer Foundation Challenge Award.

## Graphical abstract

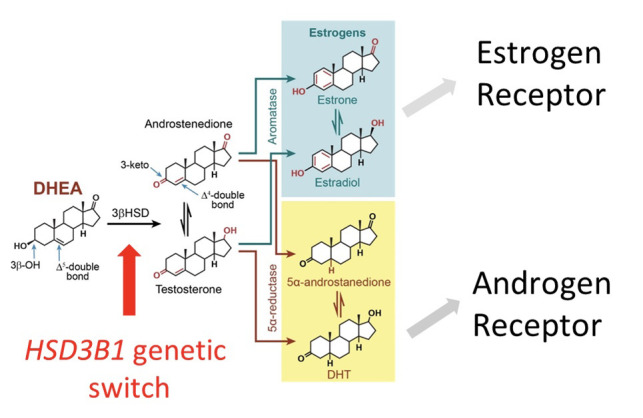

## Introduction

Estrogen exposure increases the risk of estrogen receptor–driven (ER-driven) breast cancer. Data show the effects of exogenous estrogen exposure in postmenopausal women are highly dependent on the type of steroid hormone exposure, for example with hormone therapy in menopause (1, 2). Increased endogenous estrogen exposure may similarly stimulate the development of ER-driven breast cancer (3). However, unlike germline mutations in DNA repair pathway components and certain tumor-suppressive genes, inherited genetics of estrogen synthesis has not been causally and reproducibly linked to endogenous estrogen exposure that in turn drives increased risk of breast cancer (4). This may be due to inadequate power for such studies that interrogate multiple loci, the need to account for genetic variability in geography and race, other variations in hormonal physiology that might dilute such an effect, a combination of these factors, or the absence of such a link.

**Figure 1:**
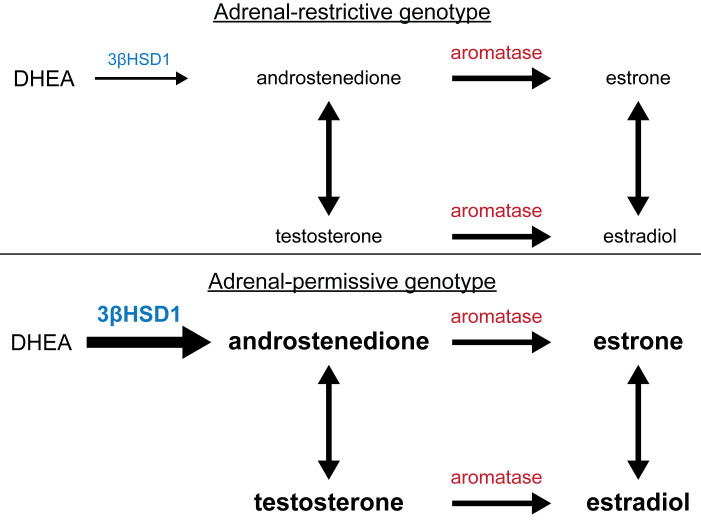
Model and mechanism for the association between adrenal-permissive *HSD3B1* genotype and ER-positive breast cancer. Comparing the adrenal-restrictive genotype (top) with the adrenal-permissive genotype (bottom), increased protein stability of *HSD3B1*-encoded 3β-hydroxysteroid dehydrogenase-1 (3βHSD1) in peripheral tissues leads to increased conversion from circulating dehydroepiandrosterone (DHEA) to androstenedione, which is subsequently converted by aromatase to estrogens. Also, 3βHSD1 is necessary for conversion of the adrenal steroid androstenediol (not shown) to testosterone.

In menopause, adrenal precursor steroids serve as the major source for peripheral metabolism and the generation of endogenous estrogens. The metabolic pathway components culminate in aromatase, which converts androstenedione to estrone and testosterone to estradiol (Figure 1) (5). The enzyme that immediately precedes aromatase is 3βHSD1, which is encoded by the *HSD3B1* gene (6). *HSD3B1* has a common, functional, germline-encoding missense variant (rs1047303) (7). The adrenal-restrictive *HSD3B1*(1245A) allele codes for an enzyme that is more rapidly degraded, whereas the adrenal-permissive *HSD3B1*(1245C) allele generates an enzyme that is more stable and enables more rapid conversion from adrenal DHEA to androstenedione and androstenediol to testosterone (8, 9). Previously, we identified the mechanistic basis for the 2 phenotypes in vitro in prostate cancer cells (7). Subsequently, at least 8 clinical studies have shown that inheritance of the adrenal-permissive *HSD3B1* allele confers worse clinical outcomes in men with advanced prostate cancer treated with medical castration because it enables conversion from extragonadal (i.e., adrenal) precursor steroids to potent androgens, i.e., testosterone and/or dihydrotestosterone (10). Consistently, inheritance of 2 copies of the adrenal-permissive allele is associated with the worst outcomes (11–14).

Given that 3βHSD1 is required for synthesis of androstenedione, the immediate precursor to estrogens and potent androgens, we wondered whether the adrenal-permissive *HSD3B1* allele was associated with endocrine system–driven cancers in the female sex. Here, we tested the hypothesis that the more rapid metabolism associated with the adrenal-permissive *HSD3B1*(1245C) allele is associated with estrogen-driven breast cancer in postmenopausal women. Our hypothesis was specific to postmenopausal women; in premenopausal women, the relative contribution of estrogens produced peripherally from adrenal precursors and therefore regulated by *HSD3B1* is much smaller because of the large pool of gonadally produced estrogens prior to menopause. Given the worse outcomes in men with prostate cancer who inherit 2 copies of the adrenal-permissive allele and the accompanying cellular metabolic phenotype, our primary hypothesis is that 2 copies of the adrenal-permissive allele (hereafter, the adrenal-permissive genotype) and any associated increase in estrogen synthesis are associated with estrogen-driven postmenopausal breast cancer. Given that the frequency of the adrenal-permissive genotype varies widely by race — C allele frequency ~0.32 in Europeans and ~0.1 or less in Africans and East Asians (15), implying homozygous adrenal-permissive genotype frequencies of ~10%, ~1%, and ~1%, respectively — we tested this hypothesis in White populations because we had sufficient statistical power.

## Results

**Figure 2:**
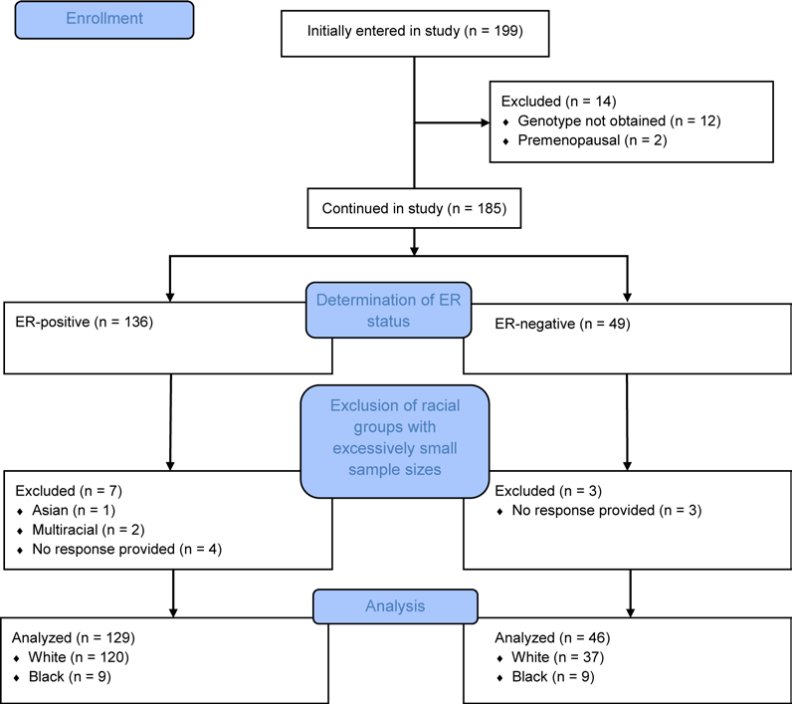
Flow diagram of study participants. From an initial pool of 199 recruited patients, 175 were ultimately included in analyses, including 157 White women (120 with ER-positive tumors and 37 with ER-negative) and 18 Black women (9 with ER-positive tumors and 9 with ER-negative).

One hundred seventy-five women who were postmenopausal at diagnosis for stage I–III, HER2-negative, invasive breast cancer with *HSD3B1* genotype were ultimately included in analyses, including 129 with ER-positive and 46 with ER-negative tumors; a flow diagram is shown in Figure 2. A cohort of 4451 patients local to the same institution was used as a general population control for the White population, of which 429 (9.6%) had the adrenal-permissive genotype. This was comparable to other studies in people of European descent and the 1000 Genomes Project (8, 10). Twenty-one of 120 (17.5%) White women with ER-positive breast cancer had the adrenal-permissive genotype, which was significantly higher than the general population (*P* = 0.0077; Table 1). The adrenal-permissive genotype was found in 2 of 37 (5.4%) women with ER-negative breast cancer. None of the 9 ER-positive or 9 ER-negative Black women had the adrenal-permissive genotype (Supplemental Table 2; supplemental material available online with this article; https://doi.org/10.1172/jci.insight.150403DS1).

Validation was sought in 2 breast cancer genomic studies. The Cambridge study characterized the genomes of 560 breast cancers with menopausal status and ER status for each tumor recorded (16). Principal component analysis (PCA) was used to infer race. TCGA genetically profiled tumors from 1098 patients with breast cancer (17). The majority of tumors were from an era prior to ER testing standardization (18). Therefore, to exclude the more equivocal cases, only those cases with documentation of at least 50% ER staining were included. The breakdown of patients included and excluded for all cohorts is in Supplemental Table 1. Altogether, the adrenal-permissive genotype was present in 28/199 (14.1%) and 28/190 (14.7%) of White postmenopausal women with ER-positive tumors in Cambridge and TCGA cohorts, respectively. In contrast, the adrenal-permissive genotype was present in 4/60 (6.7%) and 5/89 (5.6%) in ER-negative tumors in Cambridge and TCGA. In the combined validation groups, the adrenal-permissive genotype in ER-positive tumors was significantly more frequent compared with both the general population (*P* = 0.0046) and ER-negative tumors (*P* = 0.0074) (Table 1). None of 37 Black patients with ER-positive breast cancer and 43 patients with ER-negative breast cancer had the adrenal-permissive genotype. The complete breakdown of genotypes by race and ER and progesterone receptor status is in Supplemental Table 2.

In the prospective single-institution study and the TCGA validation cohort, ancestry was self-described, whereas in the Cambridge validation cohort, ancestry was determined by genetic PCA. To test whether using self-described ancestry might cause unreliable results, we reanalyzed the TCGA data using results from a study that determined genetic ancestry of all TCGA individuals (19). This only marginally changed the results (Supplemental Table 3). Using genetically determined ancestry of European rather than self-described race of White, postmenopausal patients with ER-positive breast cancer had 26/185 = 14.1% adrenal-permissive genotype, and ER-negative had 5/94 = 5.3% adrenal-permissive genotype (*P* = 0.0574 for ER-positive vs. control cohort and *P* = 0.0278 for ER-positive vs. ER-negative).

Because the hypothesis being tested was specific to postmenopausal breast cancer, enrollment in our prospective single-institution study was limited to patients who were postmenopausal at diagnosis, but we examined the adrenal-permissive genotype frequencies of premenopausal patients with breast cancer in the validation cohorts. In the TCGA cohort, White premenopausal women diagnosed with ER-positive breast cancers had a similar trend of elevated adrenal-permissive genotype frequency (9/53 = 17.0%, *P* = 0.0961 vs. control White cohort), but this was not the case in the Cambridge cohort (3/66 = 4.5%, *P* = 0.2060 vs. control White cohort). In the 2 validation cohorts together, the adrenal-permissive genotype frequencies in premenopausal White women with ER-positive (12/119 = 10.1%) and ER-negative (5/52 = 9.6%) tumors were similar to each other and to the control White cohort, but with much smaller sample sizes than for postmenopausal breast cancer (Supplemental Table 4).

**Figure 3:**
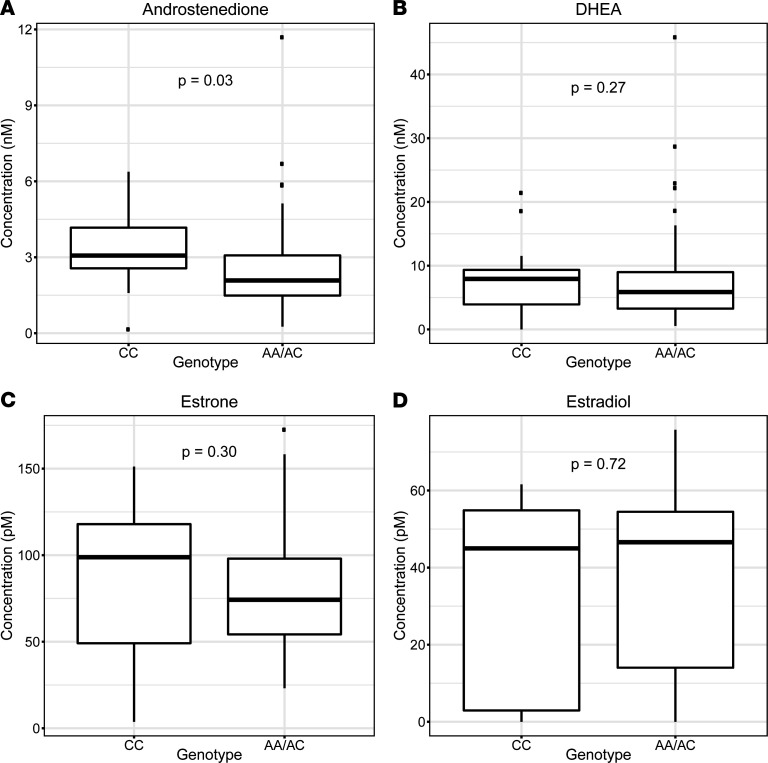
Adrenal-permissive *HSD3B1* genotype is associated with higher circulating levels of androstenedione but not of DHEA, estrone, or estradiol. Box-and-whisker plots showing circulating steroid concentrations assayed by liquid chromatography–tandem mass spectrometry from 13 women of CC (adrenal-permissive) genotype and 84 women not of CC genotype, none of whom was treated with hormonal therapies. Center lines indicate median values, boxes indicate first quartile to third quartile range, whiskers indicate values up to 1.5 times interquartile range, and points indicate outlying values. (**A**) Androstenedione concentrations. (**B**) DHEA concentrations. (**C**) Estrone concentrations. (**D**) Estradiol concentrations. Wilcoxon’s rank sum test *P* values are shown.

The adrenal-permissive genotype increased the conversion from DHEA by 3βHSD1 to androstenedione, with subsequent conversion to estrogens by aromatase (Figure 1). Circulating concentrations of DHEA, androstenedione, estradiol, and estrone were determined by mass spectrometry in 13 women who did and 84 women who did not harbor the adrenal-permissive genotype and were not treated with hormonal therapies (Figure 3). Androstenedione was significantly higher in the adrenal-permissive group compared with women who were not in the adrenal-permissive group (*P* = 0.03). DHEA, estrone, and estradiol did not differ significantly between the 2 groups.

**Figure 4:**
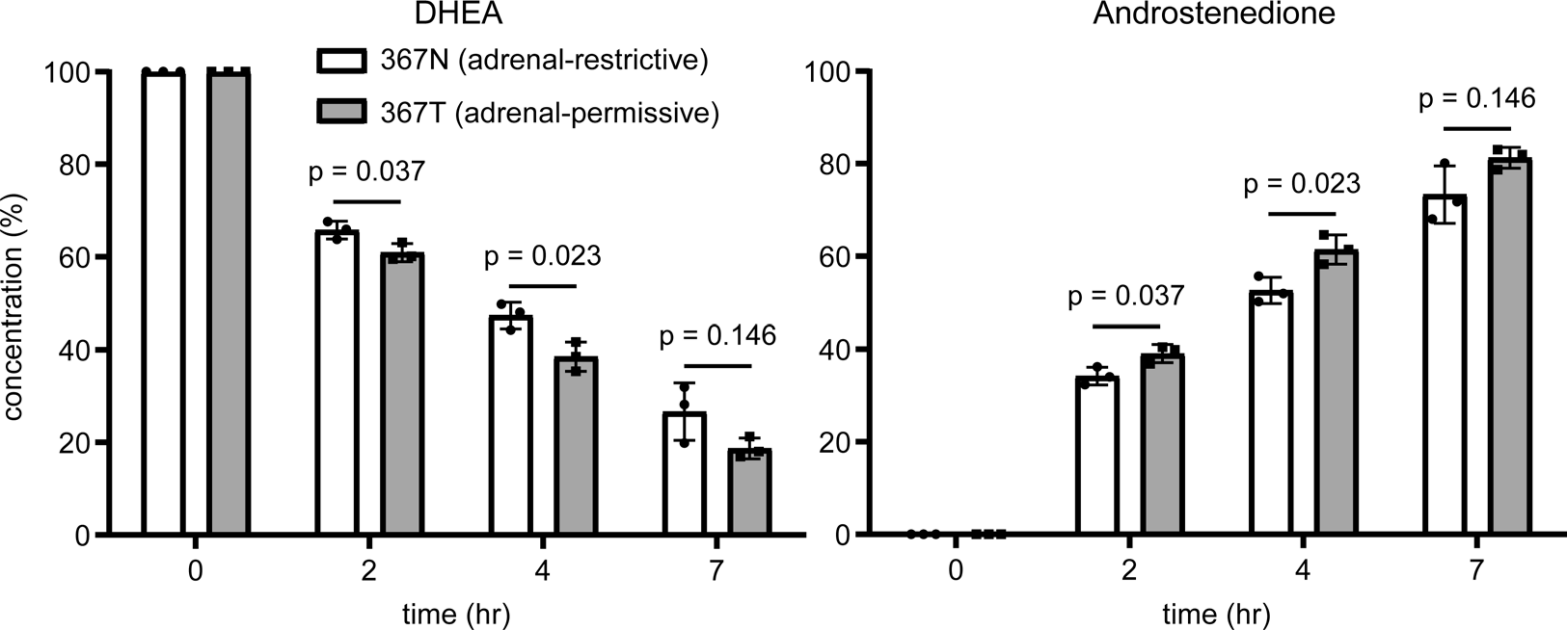
Expression of adrenal-permissive 3βHSD1(367T) in breast cancer cells results in faster conversion of DHEA to androstenedione than expression of adrenal-restrictive 3βHSD1(367N). Concentrations (percentage of total steroid) of DHEA (left) and androstenedione (right) from time 0 to 7 hours after SKBR3 breast cancer cells transfected with constructs for 3βHSD1(367N) or 3βHSD1(367T) were treated with [^3^H]-DHEA. Graphs show individual values and means ± SD from 1 experiment with biological triplicates and *P* values from 2-tailed *t* tests. Similar results were obtained in a second experiment.

We previously demonstrated in vitro that prostate cancer cells with the 367T form of 3βHSD1 encoded by the adrenal-permissive 1245C allele more rapidly convert DHEA to androstenedione than cells with the 367N form encoded by the adrenal-restrictive 1245A allele (7). To confirm that the same would occur in breast cancer cells, we expressed constructs encoding 3βHSD1(367N) and 3βHSD1(367T) in SKBR3 breast cancer cells, which we found were homozygous 1245A in our genotyping assay, and the cells were treated with DHEA. Cells expressing the adrenal-permissive 3βHSD1(367T) more rapidly converted DHEA to androstenedione than cells expressing the adrenal-restrictive 3βHSD1(367N) (Figure 4).

## Discussion

These results demonstrate that the adrenal-permissive genotype is more frequently found in women with postmenopausal estrogen-driven breast cancer compared with the general population and compared with women who have postmenopausal ER-negative breast cancer. Furthermore, the adrenal-permissive genotype associates with elevated circulating androstenedione levels. Together, these data suggest that the adrenal-permissive genotype increases risk of estrogen-driven postmenopausal breast cancer.

Our hypothesis was specific to postmenopausal breast cancer, because in postmenopausal women, the adrenals are the sole source of sex steroids, whereas in premenopausal women, estrogen synthesis from adrenally derived precursors would merely supplement the larger estrogen pool produced in the ovaries. For this reason, our prospective study did not include premenopausal women; nonetheless, we analyzed data for premenopausal breast cancer in the validation cohorts and did not find evidence for a similar effect of adrenal-permissive genotype. However, our sample sizes for premenopausal breast cancer were small, so these results should be considered preliminary.

Although our hypothesis pertained specifically to ER-positive postmenopausal breast cancer, we also included ER-negative tumors in the prospective study, because if similar trends were found in both ER-positive and ER-negative breast cancer, that would contradict our hypothesis that adrenal-permissive genotype specifically increases the risk of ER-positive breast cancer. ER-negative breast cancer is considerably less common than ER-positive, so it was more difficult to obtain sufficient statistical power to draw meaningful conclusions about ER-negative breast cancer; the observed trend was consistent with adrenal-permissive genotype being enriched in ER-positive but not ER-negative breast cancer. Intriguingly, in all 3 cohorts (prospective trial and 2 validation cohorts), there were similar trends of adrenal-permissive genotype frequencies in postmenopausal ER-negative breast cancer being lower than in the general population, the opposite result to postmenopausal ER-positive breast cancer. This suggests the possibility that there is an interaction between the adrenal-permissive genotype and ER-negative breast cancer development. Increased estrogen production in cells expressing *HSD3B1*(1245C) might drive selection for ER positivity, whereas the corresponding lack of estrogen production in cells expressing *HSD3B1*(1245A) might drive selection against ER positivity. Additional cohorts including larger numbers of ER-negative tumors should be sought out to validate this observed trend.

Although the adrenal-permissive genotype was associated with higher androstenedione in circulation, the effect size was not large, and circulating estrone and estradiol concentrations did not differ significantly by genotype. However, it is important to note that tissue steroids often differ profoundly compared with levels in circulation. A systematic review of studies on sex steroid hormone levels in circulation and in breast tissue concluded that estrogen levels are consistently found to be higher in tissue than in blood and additionally that estradiol levels are further elevated in cancerous compared with benign breast tissue in postmenopausal women (20). Furthermore, aromatase expression is known to be specifically upregulated in the breast in postmenopausal women, which may lead to changes in local estrogen levels without affecting circulating concentrations (21). Taken together, these findings suggest that enhanced androstenedione production with the adrenal-permissive genotype, in circulation and/or at the cellular level in cells expressing *HSD3B1*(1245C), could lead to local increases in breast tissue estrogen levels that may not be reflected in measurements of circulating estrogen concentrations. The effect of adrenal-permissive genotype on tissue steroid concentrations is a topic for further study.

We additionally demonstrated, in vitro, that breast cancer cells expressing the adrenal-permissive form of the enzyme more rapidly converted DHEA to androstenedione than cells expressing the adrenal-restrictive form, adding to our previous finding of the same result in prostate cancer cells (7). The difference we observed in conversion rate between the 2 forms of the enzyme was fairly modest, which is likely due to overexpression narrowing the difference. In our previous experiments in prostate cancer cells, the difference in conversion rate between cells overexpressing adrenal-permissive and adrenal-restrictive forms was much smaller than the difference between 2 cell lines that endogenously expressed (at the mRNA level) similar levels of adrenal-permissive or adrenal-restrictive *HSD3B1* (7). However, the current lack of data showing increased DHEA metabolism in breast cancer cells naturally expressing the adrenal-permissive enzyme is a limitation of our study.

A strength of our study is that we tested a single hypothesis (adrenal-permissive genotype would be associated with higher rates of postmenopausal ER-positive breast cancer) based on an established mechanism (adrenal-permissive genotype leads to increased synthesis of the estrogen precursor androstenedione). Therefore, the study does not have the issues of multiple hypothesis testing that can make it difficult to separate real results from those occurring by chance in genome-wide association studies (GWAS). In all 3 cohorts we examined, results consistent with our hypothesis were obtained. A potential weakness is that not all White populations have identical *HSD3B1*(1245C) allele frequencies, although all fall in a similar range based on comparing different European populations in dbSNP (15) and gnomAD (22). Therefore, the control cohort may not be a perfect control for all the study cohorts. On the other hand, in all 3 cohorts we observed consistent trends of both elevated adrenal-permissive genotype frequencies in patients with ER-positive tumors and lowered adrenal-permissive genotype frequencies in patients with ER-negative tumors. Any bias in the control population’s genotype frequencies would not affect the ER-positive versus ER-negative comparison, and furthermore a bias that somewhat weakened the trend for 1 ER status versus control would simultaneously somewhat strengthen the trend for the other ER status versus control.

The *HSD3B1*(1245A/C) SNP (rs1047303) has not previously been identified in GWAS for breast cancer (23). To further explore this SNP, we interrogated publicly available breast cancer GWAS results and found that in the Cancer Genetic Markers of Susceptibility Breast Cancer GWAS of postmenopausal women of European ancestry (24), rs1047303 was not found in the results, but results for 2 proxy SNPs (rs6686779 and rs3765945) each correlated with rs1047303 with *R*^2^ > 0.85 according to the National Cancer Institute’s LDproxy Tool (25) were found. Neither proxy SNP had an association with breast cancer risk in this GWAS, but because the GWAS did not break down tumors by ER status, this is unsurprising given our results. The lack of identification of rs1047303 in breast cancer GWAS despite our finding of an association in 3 independent cohorts may point to advantages of stratifying breast cancer cohorts by all 3 factors of ER status, menopausal status, and race when testing association with polymorphisms, as well as to advantages of testing a single hypothesis linked to an already-identified mechanism. The same SNP had similarly not been identified in GWAS for prostate cancer when we originally identified it (7) but has now been validated in at least 8 prostate cancer studies (10).

A potential application of these data is incorporation of *HSD3B1* genotype status into breast cancer risk models. This gene is not presently included in any genetic risk–scoring systems; however, it may add to risk prediction of estrogen-driven breast cancer. Clinical utilization of *HSD3B1* adrenal-permissive variant information could be considered at either a monogenic or polygenic level. At present, polygenic risk scores (PRSs) are not routinely used in clinical practice for discussion of risk stratification with patients. While polygenic risk is likely a significant contributor to breast cancer risk, estimated to account for 18% of familial risk (26), clinical application is limited by variability in commercially available panels, lack of consensus about variants of greatest importance, and lack of documented evidence of clinical utility with respect to breast cancer detection/outcomes. Given that the incidence of breast cancer was approximately 5%–8% higher in the ER-positive breast cancer group compared with the general population in our discovery and validation cohorts, the risk associated with this particular gain-of-function variant appears higher than that associated with variants considered for inclusion in PRS models but lower than that associated with the moderate- and high-risk genes included in hereditary breast cancer panels. Assessment of this variant independently may be important for clinicians given the hypothesized impact of this variant specifically for postmenopausal breast cancer due to the role of *HSD3B1* in adrenally mediated estrogen biosynthesis. As such, the clinical guidance provided to a premenopausal woman with this variant would likely differ from that given to a postmenopausal woman, particularly if this information is used to inform chemoprevention or screening imaging strategies.

Because well-established methods of chemoprevention with tamoxifen (27, 28) and aromatase inhibitors (29, 30) are available, identifying a population of patients with disease that is potentially enriched for response to an endocrine-based prevention approach would be clinically valuable. Such information may also be useful for patients previously diagnosed with breast cancer and now considering risks and benefits of adjuvant endocrine therapy. It has been shown that adherence to endocrine therapy is suboptimal, with up to 50% of patients stopping their prescribed endocrine therapy course before planned completion (31). Such patients are frequently worried about the risk of developing a second breast cancer, and knowing the *HSD3B1* genotype information may add to an individualized risk/benefit discussion, especially when concerns about endocrine therapy adherence arise.

When considering the factors involved in breast cancer risk, one must keep in mind not only unmodifiable risk factors, such as age and family history, but also modifiable factors that may increase exposure to endogenous estrogens, such as obesity. Lifestyle modification measures, such as weight loss, are discussed with breast cancer survivors and may have an even greater impact in patients with increased sensitivity to endogenous estrogens. Future studies of this genotype are required to determine the relative contributions of *HSD3B1* genotype versus BMI and other modifiable risk factors, particularly when investigating disease-related outcomes.

Additional directions include assessing response to various endocrine therapies in breast cancer survivors and assessment of disease-related outcomes according to adrenal-permissive genotype. Of particular interest would be differential efficacy of tamoxifen or aromatase inhibitors in the adjuvant treatment of breast cancer in women with the adrenal-permissive genotype. This may also be relevant with respect to the endocrine therapy partner selected in combination with cyclin-dependent kinase 4/6 (CDK 4/6) inhibition in the metastatic setting or in selecting which patients may be sensitive to endocrine monotherapy in the metastatic setting, as there are no biomarkers that predict differential response between endocrine monotherapy and combination endocrine/CDK 4/6 inhibitor therapy (32).

These data also surface a specific mechanism by which race and genetic ancestry may influence the timing and type of breast cancer. While the adrenal-permissive genotype is common in White women, it is rare in Black women, who also generally have a lower proportion of estrogen-driven and ER-positive breast cancer (33). On the other hand, the adrenal-permissive genotype is similarly rare in East Asian populations as in Black populations (15), but East Asian women have rates of ER-positive breast cancer more similar to those in White women (34). This is a limitation to the hypothesis that different rates of adrenal-permissive genotype contribute to different rates of ER-positive versus -negative breast cancer in patients of different ancestries and suggests that other aspects of race and ancestry also influence rates of ER positivity. Furthermore, genetic ancestry can determine the evolutionary trajectory of tumors (35). For example, it is tempting to speculate that the presence or absence of the adrenal-permissive genotype, which regulates production of the stimulus for ER, may determine the likelihood of acquiring subsequent ER-ligand-binding domain mutations as a mechanism of resistance to antiestrogen therapies (36).

Finally, these data have broad implications for genetic mechanisms that regulate general endocrine physiology. Although our prior work has established that *HSD3B1* genetic inheritance regulates peripheral synthesis of potent androgens and associated prostate cancer clinical outcomes in men absent gonadal sex steroids (8, 10), the current study suggests that *HSD3B1* is also a genetic endocrine regulator in the opposite sex and similarly regulates estrogen-driven clinical outcomes in postmenopausal women. Breast cancer is not the only female hormone-driven cancer (37), and future studies should also interrogate whether there are associations between adrenal-permissive versus adrenal-restrictive genotype and cancers of the uterus and ovary, which the current study did not address. Moreover, in support of this framework, other clinical outcomes are also associated with *HSD3B1* genetics that speak to a role for *HSD3B1* and sex steroids in inflammatory disease (38).

In conclusion, adrenal-permissive *HSD3B1* genetics is associated with ER-positive breast cancer in postmenopausal women. The results presented here should be validated in additional studies with larger sample sizes, which could also shed light on whether there are associations with more diverse breast cancer subtypes (e.g., luminal A, luminal B, Her2-overexpressing, basal) (39, 40) rather than simply classifying tumors as ER positive versus ER negative. These findings identify a mechanistic link from germline genetics of sex steroid metabolism to ER-positive breast cancer predisposition and have multiple implications for risk stratification and prevention.

## Methods

### Prospective single-institution study

Eligible patients were women with a history of stage I–III, HER2-negative, invasive breast cancer treated at a tertiary care institution. All patients were postmenopausal at cancer diagnosis, and this was confirmed with documentation of last menses at least 1 year before cancer diagnosis. For ER-positive patients, enrollment was completed before start of adjuvant endocrine therapy for purposes of serum steroid hormone analysis. However, the protocol was later amended to allow patients on active adjuvant endocrine therapy (either aromatase inhibitor or tamoxifen). Medical oncology clinics were screened by study coordinators to identify eligible patients, who were then approached for the study by their treating medical oncologist. Consent was obtained per an IRB-approved protocol, and specimens (~15 mL blood) were obtained by routine venipuncture for germline DNA extraction and circulating steroid analysis. Patient demographic information, cancer staging information, and treatment details including type of endocrine therapy received were recorded and stored in a secure REDCap database. *HSD3B1* genotyping was performed as described previously (11). Once specimen analysis was complete, *HSD3B1*(1245C) genotype information was entered into the REDCap database. A total of 199 patients were initially entered in the study, 187 of whom were successfully genotyped for *HSD3B1* (Supplemental Table 1). Among postmenopausal patients, this included 157 White women and 18 Black women; other races were excluded due to small sample sizes.

The population control for the same institution included patients recruited for elective diagnostic coronary angiography as part of the Cleveland Clinic GeneBank study (41). Peripheral blood samples were obtained, DNA was genotyped, and resulting genotype data underwent quality control and imputation as described (41). Race was self-described and 4451 White patients were identified and included, with 429 (9.6%) having homozygous adrenal-permissive (CC) genotype, 1993 (44.8%) having heterozygous (AC) genotype, and 2029 (45.6%) having homozygous adrenal-restrictive (AA) genotype. In Hardy-Weinberg equilibrium testing, the control cohort was not found to be out of equilibrium (*P* = 0.058, χ^2^ test). A comparison of demographic information for White patients in the breast cancer and control cohorts is shown in Supplemental Table 5. Although the composition of the control cohort differed from the breast cancer cohort by including men as well as marginally in average age, the control cohort was used strictly as a control for adrenal-permissive genotype frequencies in a White population local to the same institution. One would not expect a priori that adrenal-permissive genotype frequency would be affected by sex or age, nor did we find evidence for such differences (homozygous adrenal-permissive genotype frequencies in control cohort: male 299/3007 = 9.9%, female 130/1444 = 9.0%, *P* = 0.329 by Fisher’s exact test; average age ± SD for homozygous adrenal-permissive genotype 64.5 ± 11.5, for other genotypes 64.2 ± 11.1, *P* = 0.611 by 2-tailed *t* test).

### Liquid chromatography–tandem mass spectrometry analysis of adrenal androgens and estrogens in human serum

#### Steroid extraction protocol.

Serum aliquots stored at –80°C were thawed and briefly inverted multiple times. An equal volume (250 μL) of serum from each aliquot was then transferred to a borosilicate glass tube. Samples were spiked with 10 μL internal standard mix [5 ng/mL of E2-^13^C_3,_ 25 ng/mL, androstene-3, 17-dione-2,3,4-^13^C_3_ and 5α-dihydrotestosterone-d3 (16,17,17-d3)] and briefly vortexed. Methyl-tert-butyl ether, 2 mL (MTBE, Acros), was added to each tube, which was then capped and vortexed for 5 minutes using a multitube vortex mixer (Thermo Fisher Scientific) and then centrifuged for 5 minutes at 2095*g* at 4°C. After centrifugation, the samples were placed on dry ice for 15 minutes, and the extracted steroids in the MTBE fraction were collected in a new glass tube. The liquid-liquid extraction was repeated, and the combined MTBE layers were dried under nitrogen gas and then reconstituted in 150 μL 50% methanol/water (v/v). Reconstituted samples were vortexed 5 minutes on a multitube vortex mixer and centrifuged 1 minute at 2095*g* at 4°C. Samples were transferred to 1.5 mL microcentrifuge tubes and centrifuged at 15,871*g* for 10 minutes at 4°C. Each supernatant was divided in half and collected in separate HPLC vials, one for analysis by mass spectrometry of estrogens and one for analysis of androgens.

#### Instrumentation and data analysis.

Extracted steroids were quantified using stable isotope dilution liquid chromatography–tandem mass spectrometry. The liquid chromatography–tandem mass spectrometry system consisted of an ultra-pressure liquid chromatography system (UPLC; Shimadzu Corporation), composed of 2 LC-30AD pumps, a DGU-20A5R vacuum degasser, a CTO-30A column oven, an SIL-30AC autosampler, and a CBM-20A system controller coupled with a Qtrap 5500 mass spectrometer (AB Sciex).

#### Estrogen analysis.

Briefly, extracted serum samples were injected into a Shimadzu Corporation UPLC system and separated through a C18 column (InfinityLab Poroshell 120 EC-C18 column, 4.6 × 75 mm, 2.7 μm, Agilent) using a gradient starting at 20% solvent B (methanol/acetonitrile [90/10, v/v] containing 0.25 mM ammonium fluoride) increasing over 3.50 minutes to 75% solvent B; from 3.51 minutes to 11 minutes, solvent B was gradually increased to 97%, and at 11.01 minutes 100% B was run for 5 minutes (end time 16 minutes). The overall total flow rate was 0.3 mL/min, except for the period of 7.00 to 12.00 minutes, when it was adjusted to 0.15 mL/min.

Solvent A was HPLC-grade water containing 0.25 mM ammonium fluoride. The estrogens were detected on a Qtrap 5500 mass spectrometer using electrospray ionization (ESI) in negative ion mode and multiple reaction monitoring (MRM) using characteristic parent → daughter ion transitions for the specific molecular species monitored. 17β-Estradiol-2, 3, 4-^13^C_3_ (MilliporeSigma) was used as an internal standard for calibration of estrogens in human serum.

#### Androgen analysis.

Extracted steroids were injected onto a UPLC system (Shimadzu Corporation), and the androgens were separated on a C18 column (Zorbax Eclipse Plus C_18_ column, 150 mm × 2.1 mm, 3.5 μm, Agilent) using a gradient starting from 20% solvent B [acetonitrile/methanol (90/10, v/v) containing 0.2% formic acid] that was increased over 4 minutes to 75% solvent B run for 10 minutes, followed by 95% solvent B for 3 minutes. Solvent A was HPLC-grade water with 0.2% formic acid. The androgens were quantified on a Qtrap 5500 mass Spectrometer (AB Sciex) using ESI in positive ion mode and MRM. Androstene-3,17-dione-2,3,4-^13^C_3_ and 5α-dihydrotestosterone-d3 (16,17,17-d3) (Cerilliant) were used as internal standards for calibration of androgens in human serum.

Data acquisition and processing for estrogens and androgens were performed using MultiQuant (version 3.0.1) from AB Sciex. Peak area ratio of the analyte over the internal standard was used for quantification. Each sample run included calibration curves with standards for data quantification using the analyte/internal standard peak area ratio.

### Cambridge and TCGA validation

#### Cambridge validation.

Five hundred sixty patients were recruited from Australia, Belgium, France, Iceland, Italy, the Netherlands, Norway, the United Kingdom, the USA, Singapore, South Korea, and Sweden, with breast cancers of differing molecular subtype and stage (16). DNA was extracted from normal somatic tissue (peripheral blood lymphocytes, adjacent normal breast, or skin) for whole-genome sequencing (WGS) of the germline. Clinical data were recorded according to the International Cancer Genome Consortium guidelines.

Germline WGS was run using 108 base/100 base genomic paired-end sequencing on Illumina GAIIx, Hiseq 2000, or Hiseq 2500 genome analyzers, in accordance with the Illumina Genome Analyzer operating manual. Short insert paired-end reads were aligned to the reference human genome (GRCH37) using Burrows-Wheeler Aligner (v0.5.9) (42). Cancer Variants Through Expectation Maximization was used for germline SNP calling (http://cancerit.github.io/CaVEMan/). SNP array hybridization was performed using Affymetrix SNP6.0 and Affymetrix protocols. SNP copy number analysis was conducted with ASCAT (v2.1.1), generating tumor profiles of allele-specific copy numbers (43). ASCAT was also applied to WGS data, producing similar results.

Intercontinental ancestry analysis was conducted with PCA using 2318 ancestry-associated SNPs utilized by Amos et al. (44). Each patient was then plotted using the score on the first 2 PCs, and the ancestry of the resultant clusters was identified by matching the position of the 4 ancestries previously estimated by Campbell et al. (45). The 446 patients with PC1 less than 0 and PC2 less than 5 were identified as possessing European ancestry and the remaining patients were excluded. Of these patients, 259 were postmenopausal (using the definition of menopausal status or age more than 55 years).

#### TCGA validation.

Germline genotype data for patients with breast cancer were available from National Cancer Institute’s TCGA (17), specifically project TCGA-BRCA, which contains a mixture of solid tissue breast cancers for 1098 patients. Data were obtained from the NCI Genomic Data Commons Legacy Archive (17). Genotype array data (Affymetrix 6.0) for 757 documented White patients were downloaded and harmonized with the TOPMed Freeze 5 panel using a modified version of the HRC-1000G-check-bim tool (https://www.well.ox.ac.uk/~wrayner/tools/#Checking). Chromosome 1 genotypes were imputed with the University of Michigan Imputation Server (phasing with Eagle version 2.4, imputation with Minimac4 version 1.2.4, using the TOPMed Freeze 5 panel) to generate germline genotype calls for rs1047303. The majority of TCGA tumors (diagnosis made from 1988 to 2011) (17) were from pathological analysis done prior to the 2010 American Society of Clinical Oncology/College of American Pathologists guideline recommendations for ER staining (18). ER staining now requires reporting percentage positivity, and this was not reported for many of the TCGA tumors. Therefore, for ER-positive tumors, we only included tumors reported as staining at least 50% ER positive to be sure we captured estrogen-driven tumors from a time preceding current pathological standards. After excluding patients not meeting these criteria, there were 279 White postmenopausal women and 80 Black postmenopausal women (using the definition of menopausal status or age more than 55 years). Patients of other or unknown races were excluded due to small sample sizes.

### Steroid metabolism

SKBR3 breast cancer cells were provided by Ruth Keri (Department of Cancer Biology, Lerner Research Institute, Cleveland Clinic) and maintained in DMEM with 10% fetal bovine serum. For steroid metabolism experiments, approximately 100,000 cells/well were seeded in a 12-well plate with biological triplicates. Then, 12 hours later, plasmid DNA (20 ng) encoding 3βHSD1(367N) and 3βHSD1(367T), as described previously (7), was transfected into the cells using FuGENE HD Transfection Reagent (Promega); 48 hours later, [^3^H]-DHEA (~1 × 10^6^ cpm) was added, and after incubation at 37°C, media samples were collected at specified time points. Steroids were extracted using 1:1 ethyl acetate/isooctane and dried under nitrogen gas, then dissolved in 50% methanol and injected on a 1525 HPLC system (Waters Corp). Steroids were separated on a Luna 150 × 4.6 mm, 3.0 μm particle size C_18_ reverse-phase column (Phenomenex) with a methanol/water gradient at 50°C. Column effluent was mixed with Liquiscint scintillation cocktail and analyzed using a β-RAM model 4 in-line radioactivity detector (LabLogic).

### Statistics

Adrenal-permissive genotype was summarized by sex, menopausal status, and biomarker status using frequencies and percentages. Steroid measurements were summarized using median and interquartile range by patient group. Fisher’s exact test was used to compare genotype percentages between patient groups. Wilcoxon’s rank sum test was used to compare steroids between adrenal-permissive and adrenal-restrictive genotypes. All tests were 2 sided and *P* values of 0.05 or less were considered statistically significant. Statistical analysis was carried out using SAS Studio 3.7 (SAS Institute) and R version 4.0 (R Foundation).

### Study approval

The prospective study was approved by the Cleveland Clinic IRB, and written informed consent was obtained from participants.

## Author contributions

MLK contributed to study design, enrolling patients, and writing the paper; MP performed genotyping; JM contributed to study design, performing data analyses, and writing the paper; YMC designed and performed steroid mass spectrometry; XL performed the in vitro metabolism studies and reviewed the paper; WW performed statistical analyses; PSB performed genetic data analyses; FN performed genetic data analyses; AH contributed to study design; ED contributed to study design; SC contributed to study design and writing the paper; MT enrolled patients; HCFM enrolled patients; GTB enrolled patients; WHWT and SLH contributed to data for the control population; AB contributed to study design and genetic data analyses; SNZ contributed to study design and genetic data analyses; JA contributed to study design and enrolling patients; and NS contributed to study design, writing the paper, and overseeing the project. All coauthors reviewed and revised the manuscript.

## Supplementary Material

Supplemental data

ICMJE disclosure forms

## Figures and Tables

**Table 1:**
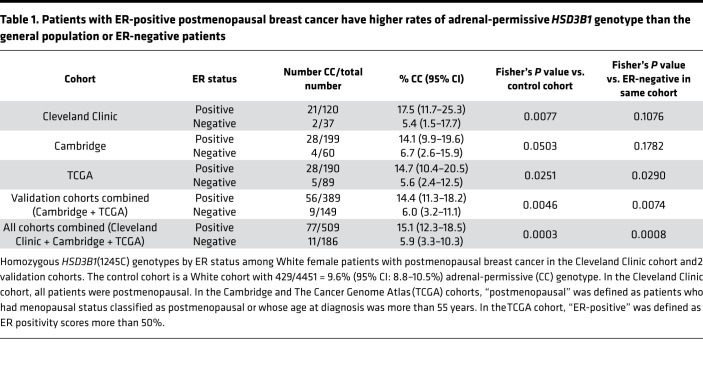
Patients with ER-positive postmenopausal breast cancer have higher rates of adrenal-permissive *HSD3B1* genotype than the general population or ER-negative patients

## Version 1 09/14/2021

In-Press Preview

## Version 2 10/22/2021

Electronic publication
