# Supplementary material for: Adrenal-permissive HSD3B1 genetic inheritance and risk of estrogen-driven postmenopausal breast cancer
Source: JCI Insight. 2021 Oct 22;6(20):e150403. doi: 10.1172/jci.insight.150403 (PMC8564898; doi:10.1172/jci.insight.150403)
Supplement: Supplemental data [file jciinsight-6-150403-s080.pdf]

Table S1. Breakdown of patients from each cohort by inclusion/exclusion criteria

Cleveland Clinic cohort

199 initial patients → 187 after exclusion of missing/discordant *HSD3B1* genotypes

**race/ethnicity**	**all genotyped patients**	**female**	**postmenopausal**
White	159	159	**157**
Black	18	18	**18**
Asian	1	1	1
Multiracial/Multicultural	2	2	2
no response provided	7	7	7
**total**	187	187	185
**total analyzed**			**175**

Cambridge cohort

560 initial patients → 555 after exclusion of missing/discordant *HSD3B1* genotypes

**race/ethnicity**	**all genotyped patients**	**female**	**postmenopausal**
White	446	442	**259**
Black	7	7	5
Asian	88	88	27
did not fall into White, Black, or Asian group by genetic PCA	14	14	5
**total**	555	551	296
**total analyzed**			**259**

TCGA cohort

1098 initial patients → 1061 after exclusion of missing/discordant *HSD3B1* genotypes

**race/ethnicity**	**all genotyped patients**	**female**	**postmenopausal**	
			**all**	**meets ER status inclusion criteria**
White	733	725	505	**279**
Black	176	173	108	**80**
Asian	59	59	33	15
American Indian or Alaska native	1	1	1	1
not available	92	91	77	10
**total**	1061	1049	724	385
**total analyzed**				**359**

In the Cleveland Clinic cohort, all patients were confirmed as postmenopausal. In the Cambridge and TCGA cohorts, not all patients had confirmed menopausal statuses; postmenopausal was defined by menopausal status of postmenopausal or age at diagnosis > 55. In the TCGA cohort, many tumors (with years of diagnosis from 1988 to 2011) were from pathologic analysis done prior to the 2010 American Society of Clinical Oncology / College of American Pathologists guideline recommendations for ER staining. ER staining now requires reporting percent positivity. Therefore, for ER-positive tumors, tumors reported as staining at least 50% ER positive were included. Race/ethnicity was self-described in the Cleveland Clinic and TCGA cohorts and determined by genetic principal component analysis in the Cambridge cohort. In the Cleveland Clinic cohort, seven subjects did not provide a response. In the TCGA cohort, the field did not contain data for 92 subjects. Numbers of patients included in paper results are bolded in last column for each cohort.

Table S2. Full *HSD3B1* genotype breakdowns among postmenopausal female breast cancer patients by cohort, race, and ER/HER2/PR status.

**Cleveland Clinic cohort**							
		***HSD3B1* genotype**					
		**AA**		**AC**		**CC**	
**race**	**status**	**count**	**%**	**count**	**%**	**count**	**%**
**White**	**ER+**	59/120	49.2	40/120	33.3	21/120	17.5
	**ER-**	20/37	54.1	15/37	40.5	2/37	5.4
	**ER+/HER2-/PR+**	50/104	48.1	34/104	32.7	20/104	19.2
	**ER+/HER2-/PR-**	8/15	53.3	6/15	40.0	2/15	6.7
	**ER-/HER2-/PR-**	19/36	52.8	15/36	41.7	2/36	5.6
	**other ER/HER2/PR**	2/2	100	0/2	0.0	0/2	0.0
**Black**	**ER+**	5/9	55.6	4/9	44.4	0/9	0.0
	**ER-**	7/9	77.8	2/9	22.2	0/9	0.0
	**ER+/HER2-/PR+**	5/8	62.5	3/8	37.5	0/8	0.0
	**ER+/HER2-/PR-**	1/1	100	0/1	0.0	0/1	0.0
	**ER-/HER2-/PR-**	7/9	77.8	2/9	22.2	0/9	0.0
**Cambridge cohort**							
		***HSD3B1* genotype**					
		**AA**		**AC**		**CC**	
**race**	**status**	**count**	**%**	**count**	**%**	**count**	**%**
**White**	**ER+**	93/199	46.7	78/199	39.2	28/199	14.1
	**ER-**	33/60	55.0	23/60	38.3	4/60	6.7
	**ER+/HER2-/PR+**	75/156	48.1	60/156	38.5	21/156	13.5
	**ER+/HER2-/PR-**	9/22	40.9	9/22	40.9	4/22	18.2
	**ER-/HER2-/PR-**	29/51	56.9	18/51	35.3	4/51	7.8
	**ER+/HER2+/PR+**	6/16	37.5	9/16	56.2	1/16	6.3
	**ER-/HER2+/PR-**	4/9	44.4	5/9	55.6	0/9	0.0
	**other ER/HER2/PR**	3/5	60.0	0/5	0.0	2/5	40.0
**TCGA cohort**							
		***HSD3B1* genotype**					

		AA		AC		CC	
**race**	**status**	**count**	**%**	**count**	**%**	**count**	**%**
**White**	**ER+**	89/190	46.8	73/190	38.4	28/190	14.7
	**ER-**	45/89	50.6	39/89	43.8	5/89	5.6
	**ER+/HER2-/PR+**	48/91	52.7	33/91	36.3	10/91	11.0
	**ER+/HER2 equivocal/PR+**	11/34	32.4	15/34	44.1	8/34	23.5
	**ER+/HER2-/PR-**	8/16	50.0	4/16	25.0	4/16	25.0
	**ER-/HER2-/PR-**	17/39	43.6	19/39	48.7	3/39	7.7
	**ER-/HER2 equivocal/PR-**	7/15	46.7	8/15	53.3	0/15	0.0
	**ER+/HER2+/PR+**	8/20	40.0	10/20	50.0	2/20	10.0
	**ER-/HER2+/PR-**	6/16	37.5	8/16	50.0	2/16	12.5
	**other ER/HER2/PR**	29/48	60.4	20/52	31.3	4/52	8.3
**Black**	**ER+**	29/37	78.4	8/37	21.6	0/37	0.0
	**ER-**	32/43	74.4	11/43	25.6	0/43	0.0
	**ER+/HER2-/PR+**	9/13	69.2	4/13	30.8	0/13	0.0
	**ER+/HER2 equivocal/PR+**	5/6	83.3	1/6	16.7	0/6	0.0
	**ER+/HER2-/PR-**	3/3	100.0	0/3	0.0	0/3	0.0
	**ER-/HER2-/PR-**	18/22	81.8	4/22	18.2	0/22	0.0
	**ER-/HER2 equivocal/PR-**	5/6	83.3	1/6	16.7	0/6	0.0
	**ER+/HER2+/PR+**	4/4	100.0	0/4	0.0	0/4	0.0
	**ER-/HER2+/PR-**	2/4	50.0	2/4	50.0	0/4	0.0
	**other ER/HER2/PR**	15/22	68.2	7/22	31.8	0/22	0.0

Counts and percentages of AA, AC, and CC genotype subjects are shown for each cohort, racial group, and ER status along with different combinations of ER/HER2/PR statuses. Totals for ER+ and ER- are shown followed by additional breakdowns by ER, HER2, and PR status. "Other ER/HER2/PR" includes subjects that did not have a HER2 status indicated along with subjects with very infrequent combinations of ER/HER2/PR statuses. In the Cleveland Clinic cohort, all subjects were postmenopausal. In the Cambridge and TCGA cohorts, postmenopausal was defined as patients who either had menopausal status classified as postmenopausal or whose age at diagnosis was > 55 years. In the TCGA cohort, ER-positive was defined as subjects with ER positivity scores > 50%.

Table S3. Using genetically determined ancestry rather than self-described race for the TCGA cohort only marginally changes the results.

cohort	ER status	number CC/total number	% CC (95% confidence interval)	Fisher's p-value vs. control cohort	Fisher's p-value vs. ER-negative in same cohort
**TCGA (self-described race = White)**	positive	28/190	14.7 (10.4 – 20.5)	0.0251	0.0290
	negative	5/89	5.6 (2.4 – 12.5)		
**TCGA (genetic ancestry = European)**	positive	26/185	14.1 (9.8 – 19.8)	0.0574	0.0278
	negative	5/94	5.3 (2.3 – 11.8)		

Comparison of homozygous *HSD3B1*(1245C) genotypes by ER status in the TCGA validation cohort among female postmenopausal breast cancer patients of self-described race White (same results as in **Table 1**) or genetic ancestry European as determined by Yuan, et al. (1).

Table S4. Adrenal-permissive genotype frequencies by estrogen receptor status in premenopausal breast cancer.

cohort	ER status	number CC/total number	% CC (95% confidence interval)	Fisher's p-value vs. control cohort	Fisher's p-value vs. ER-negative in same cohort
**Cambridge**	positive	3/66	4.5 (1.6 – 12.5)	0.2060	0.3295
	negative	2/20	10.0 (2.8 – 30.1)		
**TCGA**	positive	9/53	17.0 (9.2 – 29.2)	0.0961	0.5218
	negative	3/32	9.4 (3.2 – 24.2)		
Validation cohorts combined (Cambridge + TCGA)	positive	12/119	10.1 (5.9 – 16.8)	0.8746	1.00
	negative	5/52	9.6 (4.2 – 20.6)		

Homozygous *HSD3B1*(1245C) genotypes by ER status among white female premenopausal breast cancer patients in the two validation cohorts. The control cohort is a Caucasian cohort with 429/4451 = 9.6% (95% CI: 8.8 – 10.5%) CC genotype.

Table S5. Demographics of White subjects in institutional breast cancer cohort and control cohort.

	Cleveland Clinic breast cancer cohort, race = White (n = 157)	Cleveland Clinic GeneBank control White cohort (n = 4451)
**Sex**		
**Male**	0 (0%)	3007 (67.6%)
**Female**	157 (100.0%)	1444 (32.4%)
**Age (years) (mean ± SD)**	66.2 ± 8.6	64.3 ± 11.1
**rs1047303 C allele frequency**	0.322	0.320

The GeneBank control cohort was used as a population control for adrenal-permissive genotype frequencies in the White breast cancer cohorts and not as a control for any other characteristics of the breast cancer cohorts; therefore, differences in demographic factors that do not affect adrenal-permissive genotype frequencies should not impact the suitability of the control cohort. Within the control cohort, adrenal-permissive genotype frequencies did not appear to be affected by sex (p = 0.329) or age (p = 0.611).

1. Yuan J, Hu Z, Mahal BA, Zhao SD, Kensler KH, Pi J, et al. Integrated Analysis of Genetic Ancestry and Genomic Alterations across Cancers. *Cancer cell.* 2018;34(4):549-60.e9.
